# Functional Variants Identified Efficiently through an Integrated Transcriptome and Epigenome Analysis

**DOI:** 10.1038/s41598-018-21024-6

**Published:** 2018-02-13

**Authors:** Fanlin Meng, Guohong Yuan, Xiurui Zhu, Yiming Zhou, Dong Wang, Yong Guo

**Affiliations:** 1 School of Medicine, Collaborative Innovation Center for Diagnosis and Treatment of Infectious Diseases, Tsinghua University, Beijing, 100084 China; 2 Human Genetic Resource Center, National Research Institute for Health and Family Planning, Beijing, 100081 China; 3 National Engineering Research Center for Beijing Biochip Technology, Beijing, 102206 China; 4 Department of Basic Medicine, School of Medicine, Tsinghua University, Beijing, 100084 China

## Abstract

Although genome-wide association studies (GWAS) have identified numerous genetic loci associated with complex diseases, the underlying molecular mechanisms of how these loci contribute to disease pathogenesis remain largely unknown, due to the lack of an efficient strategy to identify these risk variants. Here, we proposed a new strategy termed integrated transcriptome and epigenome analysis (iTEA) to identify functional genetic variants in non-coding elements. We considered type 2 diabetes mellitus as a model and identified a well-known diabetic risk variant rs35767 using iTEA. Furthermore, we discovered a new functional SNP, rs815815, involved in glucose metabolism. Our study provides an approach to directly and quickly identify functional genetic variants in type 2 diabetes mellitus, and this approach can be extended to study other complex diseases.

## Introduction

Complex diseases such as cancers, coronary heart disease, hypertension, Alzheimer’s disease, Parkinson’s disease, and diabetes have a major impact on the health of human populations. They are caused by a combination of environmental and genetic factors, most of which have not yet been fully identified. The contribution of genetics, particularly the links between genetic variations and traits is a long-standing question in the study of complex diseases. Genome-wide association studies (GWAS), a powerful and popular approach to study disease-associated single nucleotide polymorphisms (SNP), have revealed a large number of genetic sites linked to disease susceptibility in complex human diseases^1^. The vast majority of significantly associated genetic variants identified through GWAS fall outside of coding regions^2,3^, complicating our understanding of how the specific SNPs increase disease susceptibility. Thus, our understanding of the role of genetic variations in disease remains limited, and it is critical to further determine their biological functions^4^.

Current strategies to screen and determine the causal risk of numerous non-coding loci have mainly utilized multi-step experimental approaches, such as identifying allelic differences in both transcriptional activity and protein-DNA binding. The allelic differences in transcriptional activity can be evaluated using luciferase reporter assays^5^. Further allelic differences in protein binding can then be analysed by electrophoretic mobility shift assay and ChIP to identify upstream transcriptional regulators^6,7^. To establish the function of a specific variant, genome editing techniques such as CRISPR-Cas9 can be applied, followed by an assessment of gene expression and cellular phenotypes^8^. In a carefully controlled experimental system, phenotypes can be compared between non-risk and risk alleles. These research strategies are widely used and have resulted in great progress towards determining the functionality of SNP candidates. However, these methods are low-throughput, slow and costly. As numerous and complex disease-associated SNPs are identified in a large number of GWAS, it is important to develop rapid, high-throughput data-driven methods to identify functional candidates for the pathogenesis of  complex diseases.

Here, we explored the involvement of various SNPs in type 2 diabetes mellitus (T2DM), for which large GWAS datasets were available. T2DM is a metabolic disease characterized by high blood glucose over a prolonged period. T2DM affects an ever-increasing proportion of the world’s population; the most recent data from the International Diabetes Federation estimates the number of people with diabetes as 415 million and the number of deaths from diabetes-related causes as ~5 million in 2015, and one in ten of the world’s population will have diabetes by 2040^9^. In China, rapid economic change has driven a dramatic shift in diets and life style, resulting in a large number of patients with diabetes^10,11^. Several risk factors for T2DM have been identified such as age, sex, body shape, physical activity and diet, as well as environmental and genetic factors^12,13^. Several studies have identified causal risk variants in the T2DM pathogenesis. Grant *et al*. found that carriers of a microsatellite within intron 3 of *TCF7L2* have a higher diabetes risk than non-carriers^14^. Claussnitzer *et al*. demonstrated that rs1421085 in intron 1 of *FTO* affects obesity risk by regulating adipocyte thermogenesis^8^. Musunuru *et al*. discovered that rs12740374 creates a specific transcription factor binding site, thereby altering hepatic expression of the *SORT1* gene^15^. Studies of these regulatory variants could deepen our understanding of the complex pathogenic process; however, a large number of SNP candidates remain to be studied. A new method to efficiently identify functional candidates is needed.

Alterations in regulatory regions are a main driver of changes in gene expression, and the mutations in a gene’s regulatory elements could have critical impacts on cellular function^16^. Furthermore, genetic variants can also modulate the histone modifications directly or indirectly^17^. Kasowski *et al*. identified genetic variants affecting histone modifications in human cells by incorporating sites of transcription factor (TF) binding and histone modification^18^. The regulatory motifs typically have location preferences, such as at the centre of H3K27ac (Histone H3 acetyl Lys27) peak^19^. The alterations in expression level of the affected genes could be measured by RNA-seq, and the regulatory regions could be identified using ChIP-seq.

With the above biological background, here we proposed a procedure called iTEA (integrated transcriptome and epigenome analysis) to identify a functional SNP candidate, by combining genetic evidence from GWAS with genome-wide maps of chromatin features (from ChIP-seq data) and gene transcription (from RNA-seq data) in T2DM, and the results were further validated using a literature search and wet experiments.

## Results

### The pipeline of integrated transcriptome and epigenome analysis (iTEA) using ChIP-seq, GWAS, and RNA-seq data

The regulatory regions are capable of modulating gene activity by increasing or decreasing the expression of specific genes. This study is based on two biological hypotheses. First, disrupting a gene’s regulatory elements could have a critical impact on cellular function^16,20^. Motifs in regulatory regions exhibit a location preference, including at the center of H3K27ac peak^19^. Here, we chose H3K27ac for ChIP-seq analysis. Second, most genes are regulated by genetic variation near to the affected genes^5^. We used RNA-seq data to provide the expression of affected genes. With the above biological background, we proposed the iTEA procedure to identify a functional SNP candidate.

**Figure 1:**
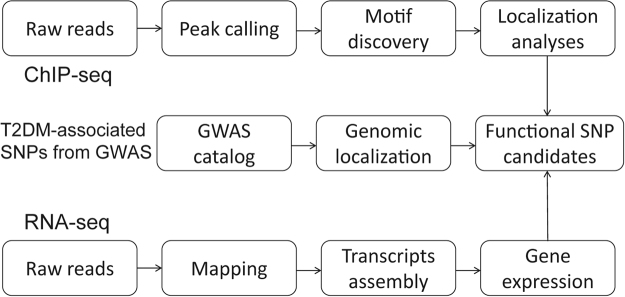
Overview of the integrated transcriptome and epigenome analysis (iTEA) for functional DNA variant identification. The pipeline of iTEA consisted of SNP collection, chromatin state characterization (ChIP-seq) and gene expression quantification (RNA-seq). To characterize the chromatin state, peak calling and motif finding were used. Then, genetic and epigenetic mapping of diabetic risk loci was implemented to prioritize and search for SNP candidates. Firstly, obtaining the T2DM SNP candidates from NHGRI GWAS catalog. Secondly, calling peaks and discovered motifs in ChIP-seq data, and aligning the motifs with SNPs to confirm whether the SNP was located in the regulatory region. Third, mapping the SNPs and assembling transcripts using FPKM for RNA-seq data to further confirm the location of functional SNP candidates, as the functional SNP can regulate the gene expression.

To describe iTEA’s procedure, we used T2DM as an example of GWAS phenotype. (a) A list of 356 T2DM-associated SNPs was obtained from the GWAS Catalog. (b) We used histone modification to identify regulatory regions in human tissues relevant to T2DM, including adipose, liver, muscle, and pancreas tissues. The Roadmap Project is a resource for ChIP-seq data. Peak calling and motif discovery were performed using HOMER to identify regulatory regions and motifs representative of DNA binding events. We mapped 356 T2DM-associated SNPs to the regulatory regions, and the overlapping SNPs with predicted DNA binding sites were reserved for the subsequent steps. (c) To validate the allele-specific expression, we required the genes flanking the overlapping SNPs to have an FPKM of at least 10. Finally, we applied iTEA to T2DM to identify regulatory variants and screened nine potential functional SNPs, of which one was further validated by luciferase assay. The processing pipeline is outlined in Fig. 1.

### The identification of functional genetic variant candidates using iTEA

**Figure 2:**
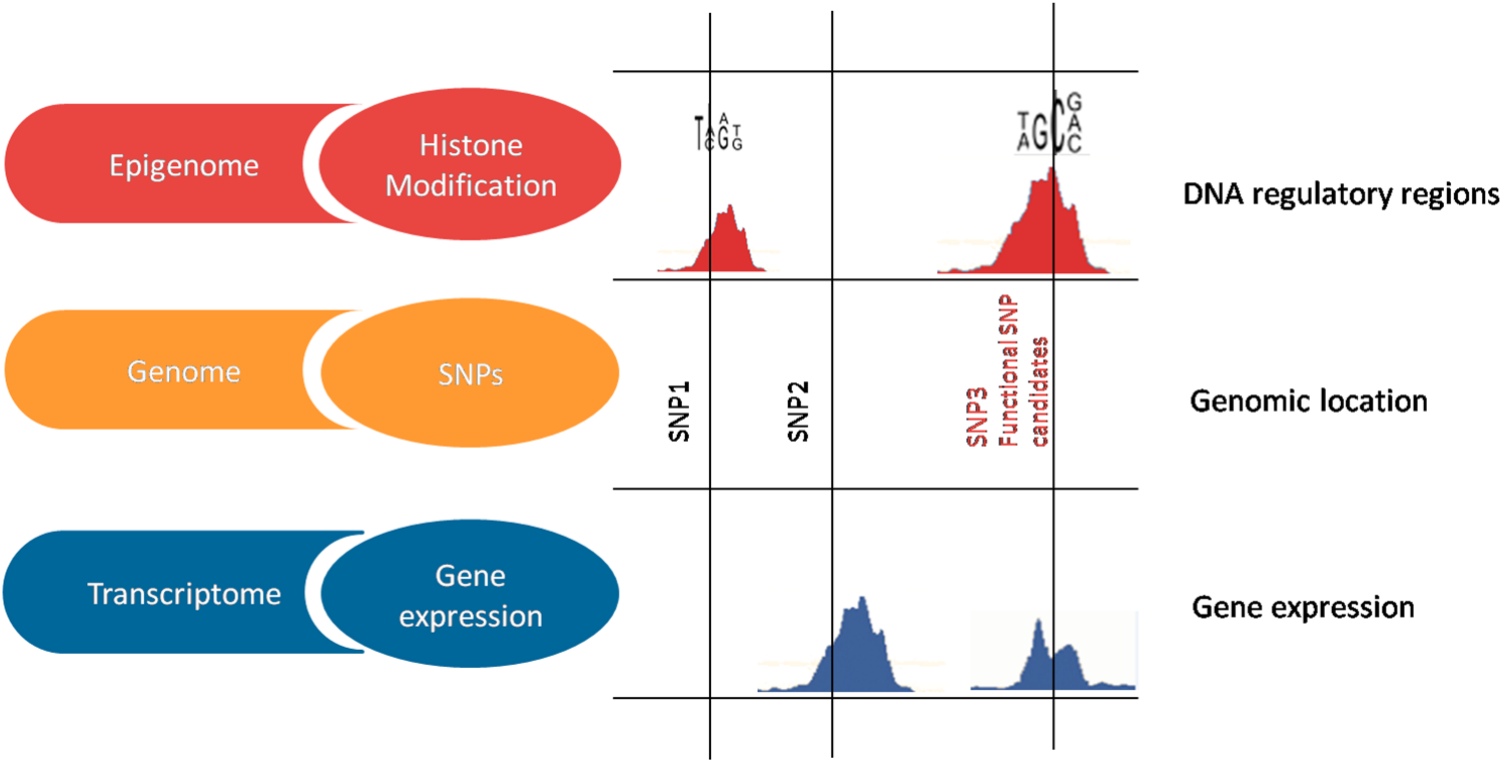
Principle of functional SNP candidates identified by iTEA using integrative  strategy. SNP3 is a functional SNP candidate, as it affects the expression of a flanking gene at the transcriptional level, and it is located in a DNA regulatory region. SNP1 and SNP2 are discarded due to lack of one of the criteria.

It has been assumed that the functional SNPs fall within gene regulatory regions and that the nearby gene is expressed. Thus, any functional SNP candidate must meet the following two criteria (Fig. 2). (1) Its genomic position coincides with a regulatory region (*i.e*. promoter or enhancer). This is supported by the notion that SNP loci may alter the binding of TFs and induce further alterations in gene expression. There exist local, proximal, or distal genetic variation-driven TF-DNA binding events^21^. ChIP-seq data is useful for identifying regulatory regions. (2) Its flanking genes are expressed in at least two of the following tissues related to T2DM pathogenesis: adipose, liver, muscle and pancreas tissues, since that most genes are regulated by genetic variation near to the affected genes^5^. To enable discovery of genes subject to proximal regulation, we captured the genes located within the region surrounding each SNP locus. RNA-seq data is then used to determine the expression levels of these flanking genes.

We compiled a list of 356 T2DM-associated SNPs as risk variant candidates. The statistical significance of *P* was set to less than 1 × 10^−5^ (Supplementary Table S1). To clearly show the location and *P* of each SNP, all 356 T2DM-associated SNPs were further analysed and displayed in a circos plot, please see Figure S1.

To identify DNA regulatory regions, we used ChIP-seq data from the Roadmap Epigenomics Projects and searched for histone modification H3K27ac signals, which marks active DNA regulatory regions, including promoters and enhancers^22,23^. For the H3K27ac ChIP-seq data for each sample, H3K27ac peaks were initially identified based on read density. We then recognized the DNA regulatory regions by identifying areas in the genome with more sequencing reads than we would expect to see by chance. Thus, putative peaks (*i.e*., non-random tag clusters of a given size) were called from the H3K27ac signals. False positive regions were removed by comparing the H3K27ac ChIP-seq signal strength in the putative peak regions with the input control signal strength. After this filtering step, proximal putative peaks were merged into final H3K27ac peaks. The filtered, merged, putative peaks indicated active DNA regulatory regions, including the binding sites of transcription factors. To determine which transcriptional factors bind at these sites, we performed motif discovery using the program HOMER^24^. Each motif enriched in these DNA regulatory regions was given a score and *P* value using the hypergeometric test. HOMER screened and returned enriched motifs with a *P* less than 0.05. The 1000 bp-window surrounding the peaks (±1000 bp from the centre of each H3K27ac peak) was used for motif discovery, allowing the identification of DNA motifs at high stringency. In addition, a 2000 bp-window surrounding the peaks was used for the identification of DNA motifs at moderate stringency.

To further confirm that these functional SNP candidates could regulate transcription, we investigated whether the expression levels of flanking genes were higher in at least two diabetes-related tissues (adipose, liver, muscle and pancreas tissues). Because T2DM affects multiple organs, gene expression in at least two of these tissues should improve confidence in the putative involvement of the SNPs. The flanking genes were selected as the nearest genes upstream and downstream relative to the transcriptional start site (TSS). If the FPKM of the  flanking genes of the functional SNP candidates is higher than 10, we considered the gene to be expressed in the corresponding tissues. This provided evidence that the functional SNP candidate may regulate transcription.

### The identification of functional genetic variant candidates at high and moderate stringency

Genic annotation of the 356 T2DM-associated variants was performed. We found that intronic and intergenic variants were the two major types (Table 1). These results are consistent with the notion that ~90% of causal variants are non-coding, as reported in an investigation of autoimmune disease^25^.

**Table 1:** Statistics of genetic annotations of 356 T2DM-assocated SNPs.

Genic region	Number of SNPs	Proportions
intergenic	151	42.42%
intronic	150	42.13%
ncRNA_intronic	19	5.34%
exonic	13	3.65%
UTR3	9	2.53%
downstream	6	1.69%
upstream	3	0.84%
UTR5	3	0.84%
fncRNA_exonic	2	0.56%

Using iTEA at high stringency, we narrowed the list of T2DM-associated variants to two possible functional genetic variants, rs35767 and rs815815 (Table 2). SNP rs35767 is an intergenic SNP and rs815815 is an intronic SNP, and both located in non-coding regions. At moderate stringency, we identified rs35767, rs815815 and seven additional functional genetic variants (rs1107366, rs10946398, rs2074356, rs2796441, rs6930576, rs4607517, and rs6937795) as candidates (Table 2).

**Table 2:** Functional genetic variant candidates provided by iTEA.

SNPID	Stringency	Genic region
rs35767	High	Intergenic
rs815815	High	Intronic
rs1107366	Moderate	ncRNA_intronic
rs10946398	Moderate	Intronic
rs2074356	Moderate	Intronic
rs2796441	Moderate	ncRNA_intronic
rs6930576	Moderate	Intronic
rs4607517	Moderate	Intergenic
rs6937795	Moderate	intergenic

To determine the regulatory effects of the 9 candidates on predicted transcription factor binding sites, we used the R package ‘motifbreakR’^26^ and HOMER to identify the response elements broken by these SNP alleles. We found that there were 58 relationships between the nine candidates and the unique predicted motifs (Supplementary Table S4). Strong disruption of these nine candidates supported their functionality.

### A well-known diabetic risk variant (rs35767) identified by iTEA at high stringency

**Figure 3:**
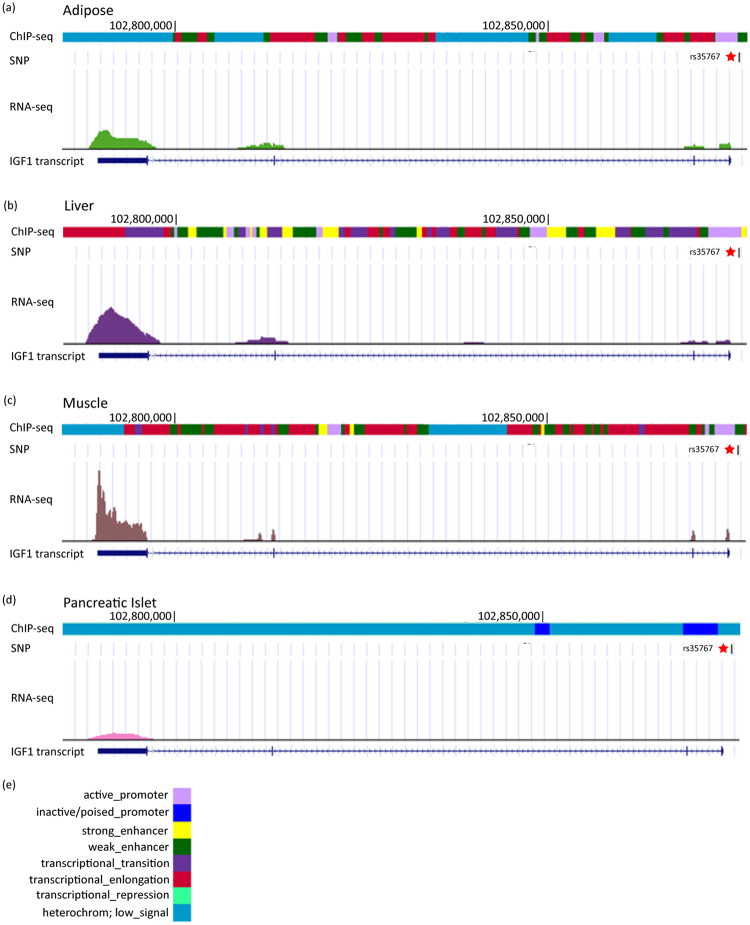
Genetic and epigenetic fine mapping of a well-known diabetic risk variant (rs35767) using iTEA. Panels a,b,c, and d show the genetic and epigenetic fine mapping of the diabetic risk SNP rs35767 in four tissue types: adipose (**a**), liver (**b**), muscle (**c**), and pancreas (**d**). We created four annotation tracks across ‘omic’ information for each tissue. The first track is a chromatin state track based on ChIP-seq data. The second is an SNP track; rs35767 is marked with a star. The third is a transcription track based on RNA-seq data. IGF1 transcript is shown for specific genomic region along with the RNA-seq and ChIP-seq signal. The diabetic risk SNP rs35767 is located in active *IGF1* promoter regions in adipose, liver and muscle samples. In accordance with the active chromatin signal, transcription is active in adipose, liver and muscle samples. Panel (e) is the legend for panels a-d. Light violet indicates active promoter regions; blue indicates the inactive promoters; yellow and green indicate the strong and weak enhancers, respectively; purple, dark pink, and cyan indicate the transcriptional transition, elongation and repression, respectively; dark cyan indicates the low-signal regions. The eight colours correspond to eight diverse chromatin states (active promoter, inactive promoter, transcriptional transition, transcription elongation, strong enhancer, weak enhancer, transcriptional repression, and heterochromatin) as defined by ChromHMM^59^ using 7 histone modification marks (H3K4me1, H3K4me3, H3K9ac, H3K9me3, H3K27ac, H3K27me3, and H3K36me3). In this study, we used H3K27ac to indicate enhancers and only assessed the influence of this histone modification across the reference transcriptome.

**Figure 4:**
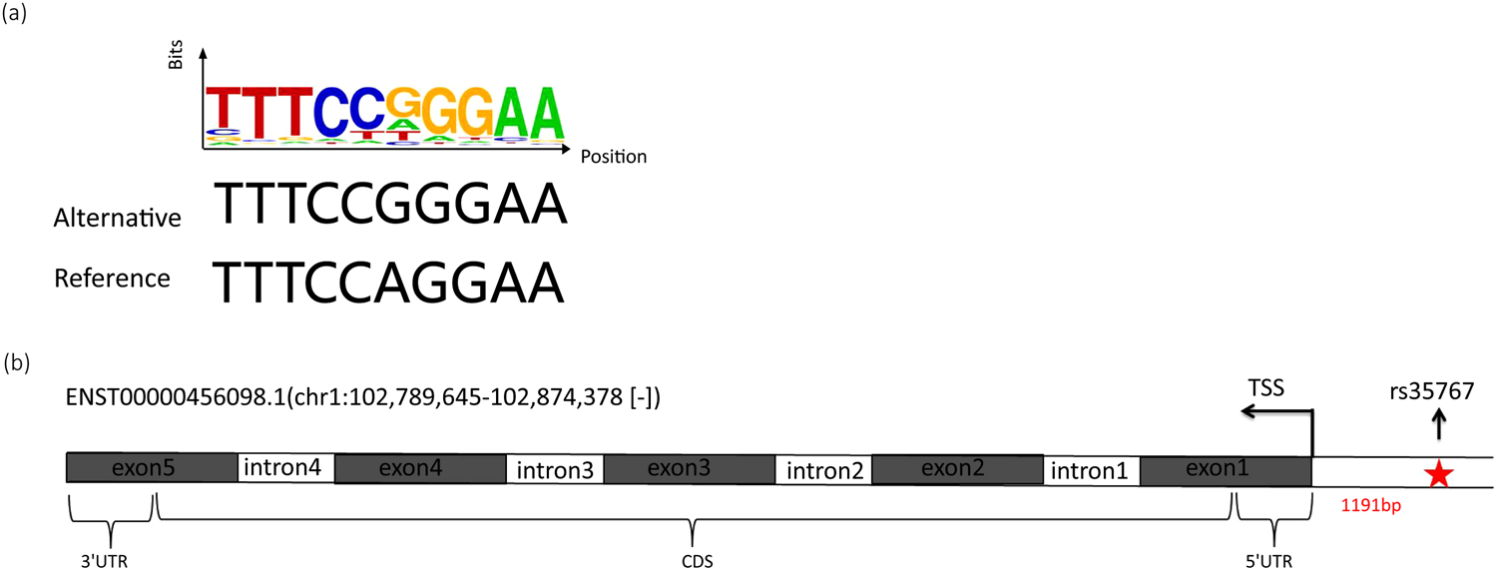
The motif structure of rs35767. Panel (**a**) illustrates the disruption of the transcription factor-binding motif by SNP rs35767. The proportions of all alternative bases are shown as relative heights of individual letters. Y-axis means bits of information content from one observation of a base at this position in a sequence, which is an indicator for the frequency of a certain base. We also show the corrsponding reference base. Panel (**b**) is the genomic structure of *IGF1* and the location of rs35767. SNP rs35767 is located 1191 bp upstream of the transcription start site of *IGF1*. CDS indicates coding sequence. UTR means untranslated region. The gray and white rectangles indicate exons and introns, respectively.

To determine whether SNP rs35767 is a functional genetic variant that regulates gene expression, we aligned the integrative transcriptomic and epigenomic maps for the region surrounding SNP rs35767 across multiple samples (*i.e*., adipose, liver, muscle and pancreas tissues). Mapping of SNP rs35767 suggested that it is located within the active promoters in three tissue types adipose, liver, and muscle (Fig. 3a–c), but not in pancreas, as the pancreas sample yielded low signal (Fig. 3d). Transcription signals were detected in adipose, liver and muscle samples, which was consistent with the chromatin signal. The motif in which rs35767 is located is shown in Fig. 4a. Based on the map of genomic loci, we found that SNP rs35767 is located 1191 bp upstream of the TSS of the insulin-like growth factor 1 (*IGF1*) gene, as shown in Fig. 4b. Our iTEA suggested that SNP rs35767 is a functional variant and may regulate *IGF1* expression by altering the regulatory regions. The regulatory effect of rs35767 on *IGF1* expression suggested by our strategy is consistent with previous results obtained by wet experiments^27,28^. This result demonstrates the feasibility of using iTEA to identify functional variants.

### A new functional SNP involved in glucose metabolism identified by iTEA at high stringency

**Figure 5:**
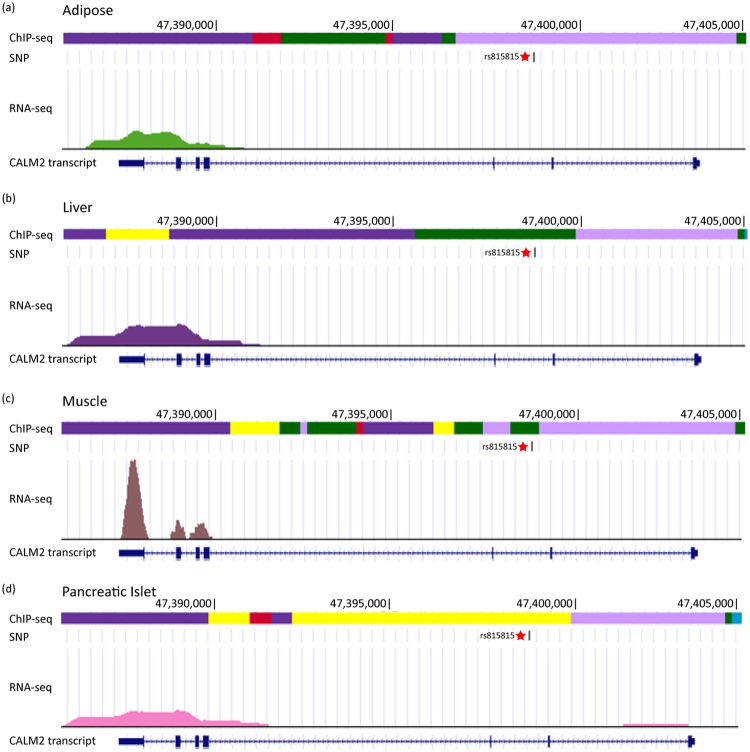
Genetic and epigenetic fine mapping of a newly characterized functional SNP rs815815 involved in glucose metabolism identified by iTEA. Figure 5–d show the genetic and epigenetic fine mapping of a newly characterized diabetic risk SNP rs815815 corresponding to four tissue types, adipose (**a**), liver (**b**), muscle (**c**), and pancreas (**d**). We created four annotation tracks across ‘omic’ information for each tissue. The first track is a chromatin state track based on ChIP-seq data. The second is an SNP track; SNP rs815815 is marked with a star. The third is a transcription track based on RNA-seq data. The diabetic risk variant rs815815 is located in *CALM2* enhancer regions in liver, muscle and pancreas samples. In accordance with the active chromatin signal, transcription is active in all samples. Panel (e) in Fig. 3e is also the legend for panels a-d in Fig. 4. Light violet indicates active promoter regions; yellow and green indicate strong and weak enhancers, respectively.

The other functional SNP identified by iTEA at high stringency was SNP rs815815. Figure 5a–d illustrates the systematic chromatin and transcriptome profiling of adipose, liver, muscle and pancreas tissues, respectively. Figure 5a shows that the SNP rs815815 was in the promoter region in the adipose tissue. In the other tissues, SNP rs815815 is located in both strong and weak enhancer elements (Fig. 5b–d). The localization of rs815815 within active DNA regulatory regions suggests that it potentially affects gene expression. SNP rs815815 is flanked by the *CALM2* gene (coding calmodulin), which is transcribed in all four tissues sampled (Fig. 5a–d). The localization of rs815815 within active DNA regulatory regions was further validated using the ChIP-seq data from the ENCODE project^29^. Both the Roadmap and ENCODE datasets indicated that rs815815 is a strong candidate as a functional SNP.

**Figure 6:**
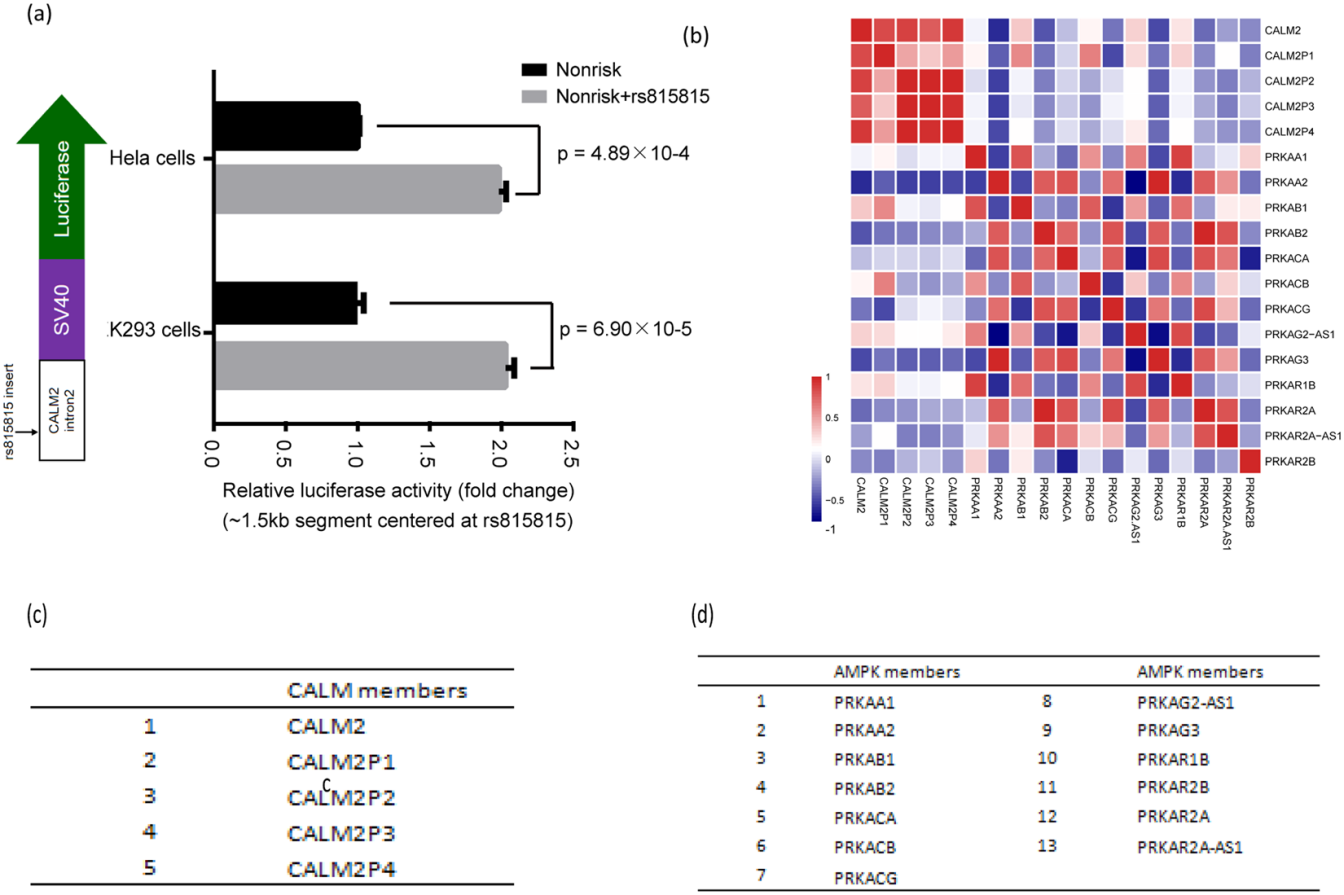
Aberrant gene expression of CALM2 caused by rs815815. (**a**) The risk allele of rs815815 (allele A) shows greater enhancer activity than the non-risk allele (allele G) in HeLa cells and HEK293 cells. The relative expression of each allele was normalized to the total expression of *CALM2* (black bars). For each cell line, the s.d. values were calculated from three independent clones for each allele. The *p* values were calculated by two-sided t-test. (**b**) CALM family genes. (**c**) AMPK family genes. (**d**) Calmodulin and *AMPK* families present negative correlation pattern in expression in tissues relevant to the development of diabetes. The value in the heatmap indicates the correlation coefficient (Pearson’s correlation).

To further assess the regulatory effect of rs815815 on *CALM2* transcription, we constructed a point mutation in the intronic region between exons 2 and 3 of the *CALM2* gene. We created allele-specific luciferase reporter constructs and measured enhancer activity in two cell lines, HeLa and HEK293. Allelic differences in enhancer activity were observed in both cell lines. The A allele resulted in significantly higher enhancer activity than the G allele (HeLa *P* = 4.89 × 10^−4^; HEK293 *P* = 6.90 × 10^−5^). Changes in allele-specific expression translated to a 1.98-fold increase in *CALM2* expression in HeLa cells and a 2.04-fold increase in HEK293 cells (Fig. 6a). Thus, the luciferase assay suggested that the risk allele (A allele) promoted the transcription of *CALM2*.

Next we sought to determine the molecular mechanism by which rs815815 and *CALM2* expression are associated with T2DM. *CALM2* is a central element in the adenosine monophosphate-activated protein kinase (*AMPK*) signaling pathway, which stimulates glucose metabolism by promoting the translocation of glucose transporter 4 (*GLUT4*)^30^. We performed a correlation analysis on the expression of calmodulin family genes and *AMPK* family genes. The correlation analysis between the expression level of *APMK* family and calmodulin was motivated by exploring the potential effects of rs815815. Calmodulin family genes and *AMPK* family genes included in the analysis are shown in Fig. 6c, d, respectively. We found that the expression levels of almost all *AMPK* and calmodulin family members were negatively correlated (Pearson’s correlation, [−0.6, 0.15], in contrast to the positive correlations observed within each gene family (Fig. 6d). It is known that *AMPK* signaling is activated in response to a variety of stimuli, including cellular stress, exercise, and a wide range of hormones. Genetic and pharmacological studies demonstrate that *AMPK* is required for maintaining glucose homeostasis, acting as a master regulator of metabolic homeostasis^31^. The luciferase reporter assay (Fig. 6a) and bioinformatics correlation analysis (Fig. 6d) suggested that when the intronic SNP rs815815 enhances *CALM2* transcription signals, *AMPK* signalling is reduced. As a consequence, *GLUT4*-mediated glucose transport is impaired, resulting in reduced glucose metabolism.

### Additional functional genetic variant candidates associated with type 2 diabetes mellitus identified at moderate stringency

**Figure 7:**
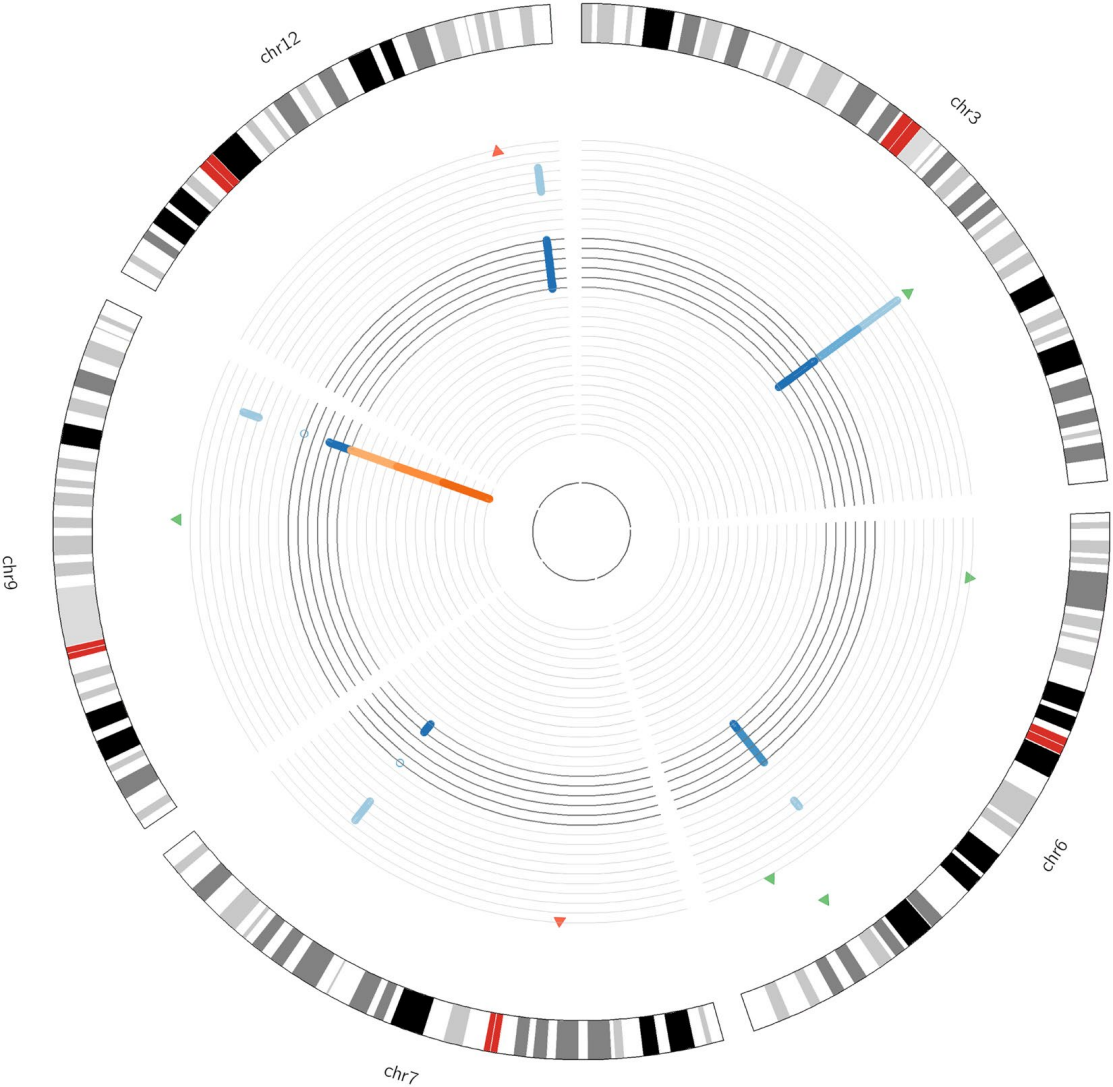
Circos plot of seven functional SNPs at moderate stringency. Circular representation of the location and *p*-value of the seven SNPs. The outermost ring represents autosome ideograms (chromosomal number is annotated), with the pter-qter orientation in a clockwise direction. The second outermost circle represents T2DM-associated SNPs and the *p*-value from GWAS. The four blue circles represent the read coverage from ChIP-seq data of four different tissues (light blue for adipose, blue for liver, dark blue for liver, very dark blue for pancreas). The four innermost, orange rings represent the read coverage from RNAseq of four different tissues (light orange for adipose, orange for liver, dark orange for liver, very dark orange for pancreas).

To identify additional putative functional genetic variants, we reanalyzed the 356 T2DM-associated SNPs using iTEA at moderate stringency. Seven additional functional genetic variant candidates (rs1107366, rs10946398, rs2074356, rs2796441, rs6930576, rs4607517 and rs6937795) were identified. Their genetic and epigenetic fine mapping is shown in Fig. 7. We further characterized the seven SNPs using a literature search in PubMed, two of the seven SNPs, rs10946398 and rs4607517, have been reported to function in T2DM.

SNP rs10946398 was identified as a candidate based on pancreatic-specific ChIP-seq peaks (Figure S2). According to our mapping results, it is located within the motif sequence AGCTGTCA (chr6: 20661034–20661042, *P* = 1 × 10^−200^). Chistiakov *et al*. showed that the minor allele (allele C) of rs10946398 (Odds Ratio = 1.21, 95% CI = 1.04–1.4, *P* = 1.6 × 10^−2^) is associated with high diabetes risk via its effect on the *CDKAL1* locus^32^. *CDKAL1* is involved in T2DM pathogenesis through impaired beta-cell function^33^ and this involvement is independent of gender, age, and body mass index^34^. He *et al*. also indicated that the genomic regions containing rs10946398 could interact with *CDKAL1*^35^.

SNP rs4607517 was also identified based on pancreatic-specific ChIP-seq peaks (Figure S3). This SNP is located within the motif sequence YSTGACAGCT (chr7: 44235667 44235677, *P* = 1 × 10^−230^). The region near rs4607517 contains the genes *GCK*, *AEBP1* and *PGAM2*. *GCK* encodes glucokinase, which is required for glucose-stimulated insulin secretion and proper glucose metabolism^36^. The genomic region containing rs4607517 has been shown to regulate *AEBP1* and *PGAM2* in IMR90 cells^37^. *AEBP1* expression was downregulated during adipogenesis^38^. *PGAM2* encodes the enzyme phosphoglycerate mutase, which is involved in a critical energy-producing process known as glycolysis. Attenuated expression of *PGAM2* led to the formation of thinner muscles in *Drosophila* embryos^39^.

### The comparison of iTEA, Haploreg and RegulomeDB

Haploreg and RegulomeDB are previously published methods to identify functional variants. HaploReg explores and visualizes the chromatin states, conservation, and regulatory motif alterations within sets of genetically linked variants^40^. RegulomeDB provides a comprehensive interface to access and visualize high-throughput, experimental data sets, as well as computational predictions and manual annotations in genome^29^.

To further validate the capability of iTEA, a set of 11 literature-validated SNPs were used as a reference set to compare the results analyzed by iTEA, Haploreg and RegulomeDB. A search of the PubMed database revealed 1942 studies involving the keywords “diabetes” and each SNP of the 356 identified in dbSNP (https://www.ncbi.nlm.nih.gov/projects/SNP/). Among them, only 449 articles mentioned at least one SNP from our list of 356 SNPs in the abstract section. We then manually catalogued 449 studies that investigated the functional or regulatory mechanisms of these SNPs. In total, we extracted 11 functional or regulatory SNPs that have been experimentally validated. The Table S2 and Figure S4a presented the results of the comparison between the three methods based on this list of 11 SNPs. For Haploreg, we considered SNPs that changed more than four motifs to be functional. For RegulomeDB, we chose SNPs with a score of less than four to be functional. By comparing the output from iTEA, Haploreg and RegulomeDB, we observed the overlaps and differences among the three methods. This result showed that iTEA was at the same level compared with the existing methods of Haploreg and RegulomeDB. This result also suggested that the existing software or bioinformatics tools could not perfectly identify functional SNPs, and it is worthwhile to complement them with new approaches. Furthermore, we used Haploreg and RegulomeDB to further analyze the nine functional candidates from iTEA. We found that six of nine functional SNPs were validated in Haploreg and five of nine functional SNPs were validated in RegulomeDB (Figure S4b, Table S3). This result further confirmed that the functional SNPs identified by using iTEA were valuable.

## Discussion

GWAS have identified numerous genetic variants associated with complex diseases, but the functions of most loci remain poorly understood. The importance of understanding the functional contributions of specific risk variants to disease pathogenesis is widely recognized. In studies of T2DM, up to 80% of the T2DM-associated loci were found to be non-protein altering variants^41^. Thus, studying the function of SNPs located in non-coding elements is important for understanding T2DM pathogenesis. Current experimental strategies for screening and identifying causal non-coding loci require multiple steps, including determining the effects of allelic differences in transcriptional activity and protein-DNA binding. Recently, a novel strategy to functionally dissect the cis-acting effect of genetic risk variants located within regulatory elements using CRISPR/Cas9 genome editing in human pluripotent stem cells has been described^42^. One post-GWAS challenge is to develop a strategy to efficiently and rapidly identity functional variants^43^.

Here, we described a data-driven bioinformatics approach to efficiently identify functional variants associated with disease risk. Compared with previous experimental strategies of studying non-coding genetic variants, iTEA can be used to directly and quickly screen genetic variants identified through GWAS. To provide more support of our findings, we further validated the iTEA results using a literature search and wet experiments. Wet experiments are essential for validating the mechanisms of selected SNPs. Nevertheless, the iTEA approach provides an efficient platform for narrowing down the list of candidate SNPs prior to functional studies.

We also extended the application of iTEA, which can be customized for the study of a specific disease by following three steps. Step (1): If GWA data is available, researchers can obtain candidate disease-associated SNPs depending on the disease of interest. The GWAS Catalog is another comprehensive resource that collects disease-associated SNPs using GWAS dating from 2008 to the present, including the majority of diseases or traits. Step (2): The regulatory regions which are necessary for regulating the expression of specific genes are required. Since that only a small percentage of the genome is responsible for coding proteins; ~98% of the genome is thought to be non-coding and is likely to have a regulatory function^44^. The regulatory regions could be identified using ChIP-seq, such as choosing H3K27ac for ChIP-seq analysis. A large amount of ChIP-seq data has been generated to provide a better annotation of the regulatory regions of the genome. Such efforts include the Roadmap Project^45^ and the ENCODE Project^46^. Individual labs are also generating ChIP-seq data increasingly^47^. Step (3): Transcriptomic data are required to measure the changes in gene expression. GTEx is a comprehensive resource that annotates the expression of transcripts (https://www.gtexportal.org/home/). GEO is an accessible resource to provide gene expression profiles generated via microarray or sequencing (https://www.ncbi.nlm.nih.gov/geo/).

Our iTEA approach successfully identified rs35767, a diabetic risk variant. This result was consistent with a meta-analysis of 21 GWAS, which suggested that rs35767 is a common variant strongly associated with glycaemic traits. Under normal physiological conditions, *IGF1* interacts with insulin to maintain normal blood glucose levels and to modulate carbohydrate and lipid metabolisms^48^. In T2DM patients, SNP rs35767 decreased the levels of *IGF1* expression, in GG genotype subjects compared with control subjects carrying A allele^28^. The reduction in insulin compared with normal physiological levels did not meet the demand to normalize glycemia, leading to ectopic glucose metabolism.

Using iTEA, we successfully discovered a new functional variant, rs815815, and demonstrated its effects on *CALM2* expression. Allele-specific regulation of *CALM2* expression was validated using a luciferase reporter assay. Previous research has suggested that *CALM2* is differentially methylated in diabetic rats^49^. These data indicated that rs815815 contributes to T2DM susceptibility by increasing *CALM2* expression.

Using iTEA, we identified additional functional genetic variants at moderated stringency. Seven additional SNPs (rs1107366, rs10946398, rs2074356, rs2796441, rs6930576, rs4607517 and rs6937795) were identified, three of which (rs10946398, rs2074356, and rs4607517) have been previously reported to be functional in T2DM.

Computational models (e.g., random forest, support vector machines, and artificial neural networks) have been widely used to determine the DNA regulatory regions, and a large number of samples are needed to obtain robust results^50–53^. In our study, we analyzed only nine samples which were insufficient for robust predictions using the existing computational models. Here, we obtained meaningful results using the nine samples. By utilizing paired ChIP-seq data in combination with RNA sequencing data for adipose, liver, muscle and pancreas tissues, it is possible to improve the quality by increasing the number of samples. In this study, we only used ChIP-seq data for the histone modification H3K27ac. Future studies will be improved by combining other data types (different histone modifications, DNase I hypersensitivity, and DNA methylation) with H3K27ac modification to identify the DNA regulatory regions to improve confidence in the pipeline. In this study, we used 1–2 kb flanking regions around the TSS in motif discovery^54^, because these regions were ranked highly for discovering transcription factor binding sites. However, this approach cannot identify transcription factors that appear in super-enhancers, which could be 100 kb long, indicating the limitation of this approach. Additionally, we used T2DM as a disease model and proposed the concept of iTEA, as this study only analyzed SNPs associated with T2DM. However, iTEA can be generalized to other diseases by analyzing different tissues and SNP candidates, as well as by altering the cut-off values.

We compared iTEA with the two commonly used tools HaploReg and RegulomeDB. We evaluated the bona fide and false positive of functional SNPs by using two common sets of SNPs. The first list of SNPs is comprised of 11 functional SNPs based on experimental evidence obtained via literature search (Table S2). The second list of SNPs comprises the nine functional SNPs identified by iTEA (Table S3). Here, we used the common SNP list to scrutinize and compare the output of three different algorithms (iTEA, Haploreg and RegulomeDB). Using this approach, we observed overlaps and differences of output among the three methods (Figure S4a, and Figure S4b). Collectively, this study suggested that determining the functionality of SNPs should be handled with care and that ideally several methods should be combined to achieve better predictions. This analysis also indicated the value to develop new approaches to complement current ones.

In summary, we proposed a new strategy termed integrated transcriptome and epigenome analysis (iTEA) for studying potential functional variants located in non-coding regions. The well-known diabetic risk SNP rs35767 was identified by iTEA, and a novel functional SNP rs815815 involved in glucose metabolism was discovered. SNP rs815815 is functionally linked with diabetic phenotypes via its regulation of *CALM2*, which acts in the *AMPK*-mediated glucose metabolism-regulation pathway. Our study established a new approach to functionally connecting genetic variation with disease-relevant phenotypes. This approach can be extended to the studies of risk loci associated with other diseases.

## Methods

### Collection of T2DM susceptibility variants

We used the NHGRI GWAS Catalog^55^ (available at www.genome.gov/gwastudies. accessed Jun. 25th, 2016) to obtain a list of GWAS associations. SNPs had been catalogued to show genome-wide association (*P* < 1 × 10^−5^) with fasting glucose, obesity, and body mass index. We obtained a list of published SNPs from the NHGRI GWAS Catalog by searching with the keyword ‘type 2 diabetes’ to retrieve primary associations and records. After filtering by *P* < 1 × 10^−5^, 520 records were obtained. Since one SNP could be associated with multiple diabetic traits or several publications, only 356 unique SNPs were included. A total of 356 variants satisfied these criteria and were termed ‘T2DM-associated SNPs’ (Supplementary Table S1).

### ChIP-seq data analysis

We obtained all available ChIP-seq data involving the adipose, liver, muscle and pancreas - four tissues related to T2DM pathogenesis - from NIH Roadmap Epigenomics Project (http://www.roadmapepigenomics.org). We obtained one sample (GSM906394) originating from adipose tissues and two samples (GSM1112808 and GSM1112809) from livers. We obtained three samples (GSM1127171, GSM1013130 and GSM910556) from muscles and two samples (GSM1127061 and GSM1127071) from the pancreases. The epigenetic data in primary human cells/tissues was obtained from Broad Institute of MIT and Harvard, UCSF, UCSD, University of Washington (Seattle) and Baylor College of Medicine. Each of the samples was obtained from an unrelated healthy subject. We performed peak analysis and motif analysis for H3K27ac modifications in each sample. Peak analysis and motif analysis were performed using HOMER (v4.6). First, the ChIP-seq data were transformed into a platform-independent data structure using the makeTagDirectory function. The results for all samples from the same tissue were merged into one data set. Second, the dataset was analysed using HOMER’s findPeaks function (using the ‘-style histone’ option) to identify peaks (regions of the genome where more reads were present than random). Following the primary, putative peak calling step (the putative peak size was set at 500 bp), three types of filters in the function were applied to compare with the Input controls. The Input control is the DNA not precipitated in ChIP-seq experiments. Third, the identified peaks were analysed using HOMER’s findMotifsGenome function to identify the *de novo* motifs of lengths 8 and10 bp. Finally, the identified peaks were annotated using the annotatePeaks.pl function.

### RNA-seq data analysis

We further obtained all available the RNA sequencing data involving adipose, liver, muscle and pancreas tissues from the NIH Roadmap Epigenomics Project. FPKM (fragments per kilobase of exon per million fragments mapped) values were used to quantify the expression of all human transcripts. FPKM values were calculated using Cufflinks v2.1.1^56^. The mapped sequence reads (bed format) were first transformed to bam format and then used as input for Cufflinks. The default parameters were used in transcript quantification. The FPKM value was calculated as follows:

1 $$FPK{M}_{i}=\frac{C}{(\frac{L}{{10}^{3}})(\frac{N}{{10}^{6}})}\,$$

Herein, *C* is the number of fragments mapped to transcript *i*, *N* is the total number of sequenced and mapped fragments, and *L* is the length of the exons of encoded by transcript *i*. Three samples (GSM1010958, GSM1120304, and GSM1120305) of adipose tissue were obtained, and the three FPKM values for each transcript were averaged to represent the expression level in the adipose tissue. We obtained one sample (GSM1157105) of the pancreas. Three samples (GSM1010968, GSM1120310, and GSM1120311) of muscles were obtained, and the three FPKM values of each transcript were averaged to represent the expression level in the muscle. We obtained two samples (GSM1067795 and GSM916093) of the liver, and the two FPKM values of each transcript were averaged to represent the expression level in the liver. Each of the samples was obtained from an unrelated healthy subject.

### Identification of functional variant candidates

To select the functional variant candidates, we aligned T2DM-associated SNPs with DNA motifs found in H3K27ac peak regions using BEDTools^57^. The BEDTools command intersectBed command was used with the parameters -a, -b, -wa, -wb. Considering the effects of transcriptional regulation of the functional variant candidates, we used a higher FPKM (>10) to select the functional variant candidates. All RNA-seq and ChIP-seq annotations of SNP loci were displayed in the UCSC Genome Browser^58^.

### Correlation analysis

Correlation analysis was applied to determine the direction and strength of correlations in gene expression between genes in the *AMPK* and calmodulin families, using RNA-seq data obtained for the four aforementioned tissues. We used the Pearson correlation coefficient to measure the linear correlation between the expression level of *AMPK* and calmodulin. The value of the Pearson correlation coefficient ranges from -1 to 1, in which 1 is total positive linear correlation, 0 is no linear correlation, and -1 is total negative linear correlation. The Pearson correlation coefficient was calculated using the cor() function and is illustrated using the pairs() function coupled with a custom R function of panel.cor(). Details were shown in the Supplementary Information. The correlation matrix was applied to illustrate the heatmap using R package ‘pheamap’. The samples used for the correlation include one sample (GSM1157105) from pancreases, three samples (GSM1010968, GSM1120310, and GSM1120311) from muscles, and two samples (GSM1067795 and GSM916093) from livers. Each of the samples was obtained from an unrelated healthy subject by the Broad Institute, UCSF, UCSD, University of Washington, or Baylor College of Medicine.

### Assembly of reporter constructs

We prepared a 951 bp genomic DNA fragment containing the human SNP rs815815, located in intron 2 of the human CALM2 gene. The DNA fragment was amplified by PCR from human genomic DNA using the following primers:

5′-GAAAATAAACTACTTTCTGGATTCCTTCTTGAATTTTC-3′ (forward primer); 5′-AATCCAGAAAGTAGTTTATTTTCCCTGCTCAACAATTT-3′ (reverse primer). The fragment was inserted into the pGL3-Promoter (pGLP) reporter vector (Promega) to create the pGLP-G construct using Hieff CloneTM One Step PCR Cloning Kit (Yeasen) according to the manufacturer’s protocol. The SNP locus contained the G allele in the aforementioned construct. Site-directed mutagenesis was used to modify the pGLP-G construct into the pGLP-A construct in which the SNP locus contained the A allele. Mutagenesis was conducted by overlapping PCR, and two pairs of primers were designed as follows.

Primer pair 1: 5′-GGTAAAATCGATAAGGATCCGCACAACAACCCTGCAAGGTAAG-3′ (forward primer);

5′-AATTCAAGAAGGAATCCAGAAAGTAGTTTATTTTCCCTGCTCAACAATTT-3′ (Mutagenic reverse primer: mutation site, underlined);

Primer pair 2: 5′-ATTGTTGAGCAGGGAAAATAAACTACTTTCTGGATTCCTTCTTGAATTTT-3′ (Mutagenic forward primer: mutation site, underlined);

5′-TCTCAAGGGCATCGGTCGACGGCTAGGGTTAAGTGGGATTGGG-3′ (reverse primer).

### Cell culture and luciferase reporter assay

Two independent clones for each allele were verified by sequencing and were transfected into HeLa and HEK293 cell lines in biological triplicates. The cells were cultured in high-glucose DMEM (Gibco) supplemented with 10% foetal bovine serum (Gibco). The cells were maintained at 37 °C in a humidified 5% CO_2_ atmosphere. The cells were inoculated in 24-well plates at 4 × 10^4^ cells/well and were grown to 70–80% confluence, and then were co-transfected with 0.5 μg of either pGLP-A or pGLP-G, and 0.05 μg control vector (Promega) using FuGENE® HD Transfection Reagent (Promega) according to the manufacturer’s instructions. The cells were harvested and lysed 48 h after transfection. Firefly and Renilla luciferase activities were measured with a Synergy H1 Hybrid Reader (BioTek). Renilla luciferase activity was used as an internal control.

### Literature-based validation

We performed a comprehensive literature search to see which of the 356 T2DM-associated variants have been identified as functional variants. We retrieved 1942 articles from PubMed using the keywords of “diabetes” and IDs of SNPs in dbSNP (https://www.ncbi.nlm.nih.gov/projects/SNP/). Among them, we manually selected 449 articles that mentioned at least one SNP from our list of 356 SNPs. In the 449 articles, 11 SNPs were validated to be functional variants with experimental approaches.

### Availability of data

NHGRI GWAS catalog provides (www.genome.gov/gwastudies) the list of GWAS association from previous investigations. ChIP-seq and RNA-seq datasets are available from the Roadmap Epigenetics Project (http://www.roadmapepigenomics.org). The data after our processing is available upon request.

## Electronic supplementary material

Supplementary Information
