# Supplementary material for: Functional Variants Identified Efficiently through an Integrated Transcriptome and Epigenome Analysis
Source: Sci Rep. 2018 Feb 13;8:2959. doi: 10.1038/s41598-018-21024-6 (PMC5811556; doi:10.1038/s41598-018-21024-6)
Supplement: Supplementary file 1 — Supplementary Information [file 41598_2018_21024_MOESM1_ESM.pdf]

Functional Variants Identified Efficiently through an Integrated Transcriptome and Epigenome Analysis

Fanlin Meng, Guohong Yuan, Xiurui Zhu, Yiming Zhou, Dong Wang, Yong Guo

Supplementary Information

Supplementary Methods

Supplementary Figures

Supplementary Figure S1. The circos plot of 356 T2DM-associated SNPs and their *p*-value.

Supplementary Figure S2. The genetic and epigenetic fine mapping of SNP rs10946398.

Supplementary Figure S3. The genetic and epigenetic fine mapping of SNP rs4607517.

Supplementary Figure S4. The comparison of iTEA, Haploreg and RegulomeDB using two set of SNPs.

Supplementary Tables

Supplementary Table S1. The list of 356 T2DM-associated SNPs.

Supplementary Table S2. The iTEA, Haploreg and RegulomeDB scores of the 11 literature-validated SNPs.

Supplementary Table S3. The RegulomeDB and Haploreg scores of the nine iTEA-identified SNPs.

Supplementary Table S4. The identified response elements of the nine functional SNP candidates from iTEA.

Supplementary Methods

Identification of the response elements broken by SNPs

The R package of ‘motifBreakR’[1] allows researchers to assess the effects of variants on predicted transcription factor binding sites. motifbreakR implies that whether the sequence surrounding a variant is a good match, and how much information is gained or lost in one allele of the variant relative to the other. We applied 'motifbreakR' to explore the response elements for our list of SNPs, all predicted motifs were obtained from HOMER. The input file was the list of SNP indicated by rs number. The default parameters were used.

Correlation analysis

We used Pearson's correlation coefficient, which is commonly represented by ρ (rho). Here, X (or Y) represents a vector of expression level of a member of the AMPK (or calmodulin) family across multiple samples. The formula for ρ[2] is:

$$\rho_{X,Y} = \frac{\mathrm{cov}\,(X,Y)}{\sigma_X \sigma_Y},$$

where

$$\mathrm{cov}(X,Y) = E[(X - \mu_x)(Y - \mu_Y)]$$

The greater the degree of negative correlation, the closer the value of ρ is to -1. In our analysis, ρ was -0.6, so we concluded that calmodulin was negatively correlated with AMPK. The Pearson correlation coefficient was calculated using the cor() function and illustrated using the pairs() function coupled with a custom R function of panel.cor() as follows.

```
panel.cor <- function (x, y, digits=2, prefix="", cex.cor,)
{
      usr <- par("usr"); on.exit(par(usr))
      par(usr = c(0, 1, 0, 1))
      r <- cor(x, y)
```

```
    txt <- format(c(r, 0.123456789), digits=digits)[1]
    txt <- paste(prefix, txt, sep="")
    if(missing(cex.cor)) cex.cor <- 0.8/strwidth(txt)
    text(0.5, 0.5, txt, cex = cex.cor * r)
}
```

Reference

1. Coetzee, S. G., Coetzee, G. A. & Hazelett, D. J. motifbreakR: an R/Bioconductor package for predicting variant effects at transcription factor binding sites. *Bioinformatics* **31**, 3847-3849 (2015).
2. Zaiontz, C. (2015). "Real Statistics Using Excel: Correlation: Basic Concepts, retrieved 2015-02-22.".

Supplementary Figures

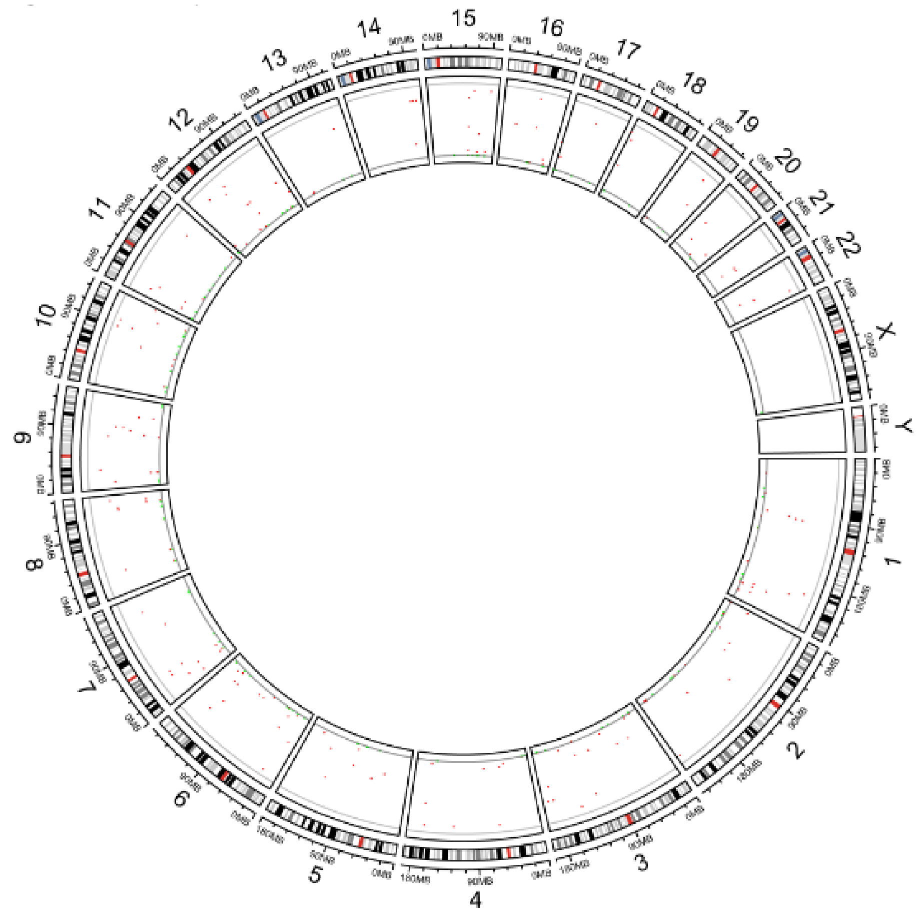

Figure S1. The circos plot of 356 T2DM-associated SNPs and their p-value. The circos plot shows the location and *P* of each SNPs in the list of 356 T2DM-associated SNPs. Each red dot represents a genomic locus of one SNP. The inner circle indicates the *P* value. The *P* value is smaller when it is closer to the center of the circle.

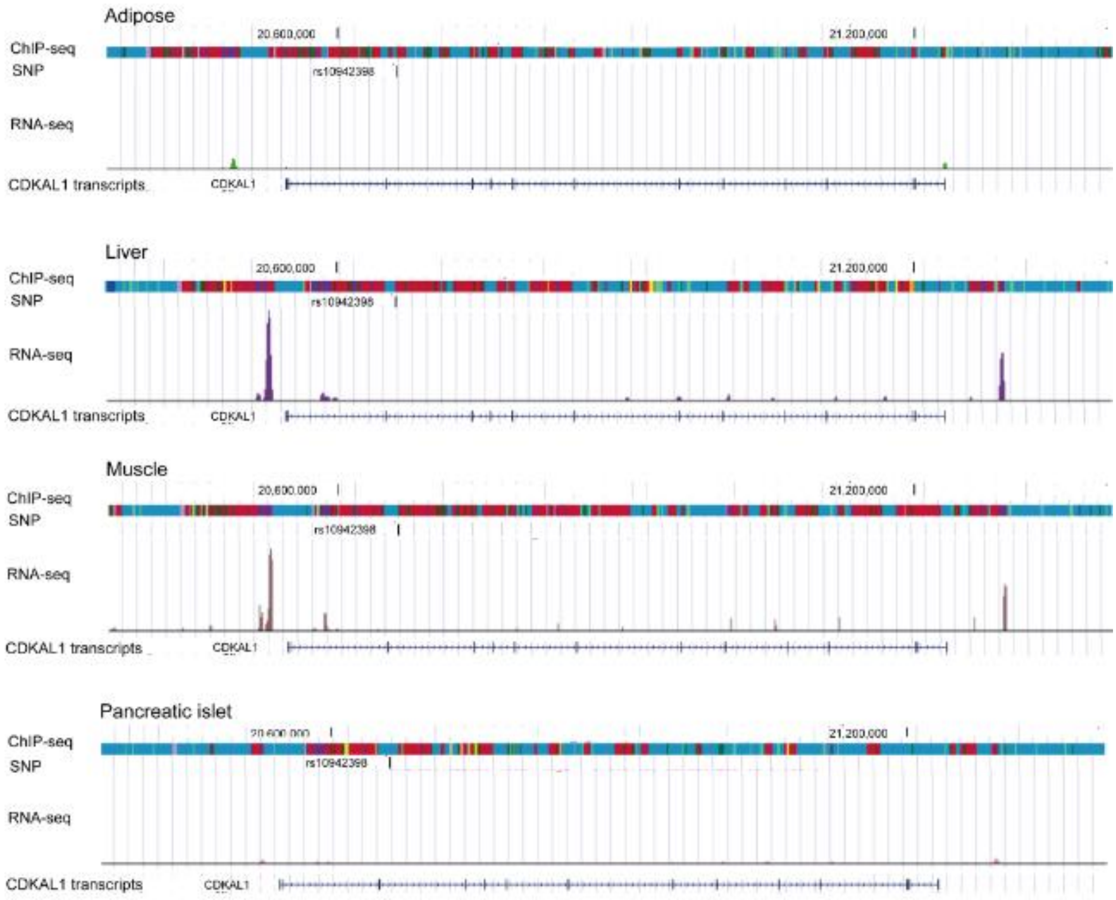

Figure S2. The genetic and epigenetic fine mapping of SNP rs10946398. Genetic and epigenetic fine mapping of SNP rs10946398. Four annotation tracks across 'omic' information for each tissue are shown. The first track is chromatin state track based on ChIP-seq data. The second is SNP track. The third track is transcriptomic track based on RNA-seq data. The fouth track indicates the reference transcript. The legend is the same as in Figure 3e. Red indicates transcriptional enlongation. Purple indicates transcriptional transition.

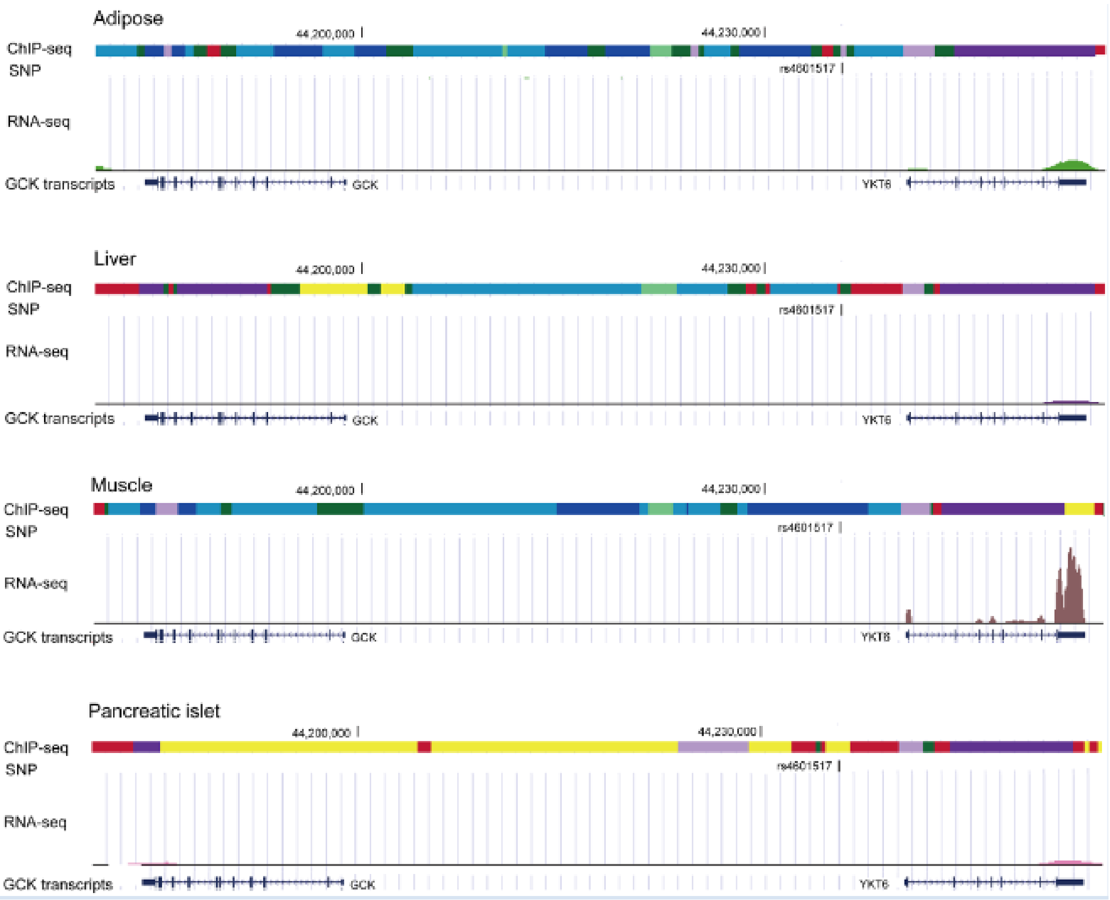

Figure S3. The genetic and epigenetic fine mapping of SNP rs4607517. Four annotation tracks across 'omic' information for each tissue are shown. The first track is chromatin state track based on ChIP-seq data. The second is SNP track. The third track is transcriptomic track based on RNA-seq data. The fouth track indicates the reference transcript. The legend is the same as in Figure 3e. Red indicates transcriptional elongation. Purple indicates transcriptional transition. Yellow and green indicate strong and weak enhancers, respectively.

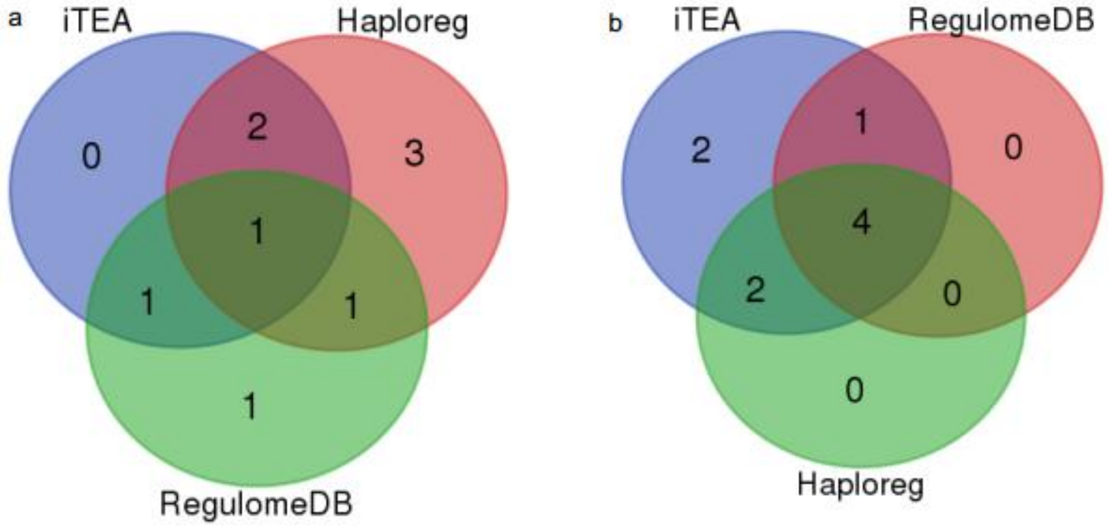

Figure S4 The comparison of iTEA, Haploreg and RegulomeDB using two sets of SNPs. **(a)** The 11 literature-validated SNPs were used as a reference set to be analyzed by iTEA, Haploreg and RegulomeDB. The result showed that four, seven and four of the 11 SNPs were further validated by iTEA, Haploreg and RegulomeDB, respectively. **(b)** The nine functional SNP candidates re-analyzed by Haploreg and RegulomeDB**.** There are nine functional SNPs identified using iTEA. Among the nine functional candidates, six SNPs were further validated by Haploreg and five SNPs were validated by RegulomeDB, respectively.

Supplementary Tables

Table S1. The list of 356 T2DM-associated SNPs.

dbSNPID	Chr	Start	End	P-value
rs10794657	1	24626174	24626174	2.00E-07
rs10911021	1	182081960	182081960	2.00E-08
rs10923931	1	120517959	120517959	4.00E-08
rs11165354	1	92194322	92194322	4.00E-06
rs12027542	1	233340154	233340154	4.00E-07
rs1497828	1	217527024	217527024	4.00E-06
rs16844716	1	199356516	199356516	9.00E-06
rs17045328	1	207652176	207652176	7.00E-06
rs17106184	1	50909985	50909985	4.00E-09
rs17110736	1	94462769	94462769	5.00E-06
rs1751492	1	65992625	65992625	6.00E-13
rs2075423	1	214154719	214154719	2.00E-06
rs2791553	1	219676042	219676042	5.00E-07
rs2811893	1	59162148	59162148	3.00E-07
rs2820446	1	219748818	219748818	2.00E-06
rs3001032	1	219727779	219727779	4.00E-08
rs340874	1	214159256	214159256	7.00E-12
rs35214987	1	88226329	88226329	9.00E-07
rs4077468	1	205914757	205914757	1.00E-09
rs478093	1	120255126	120255126	2.00E-14
rs6426514	1	228880115	228880115	2.00E-06
rs7542900	1	95070041	95070041	6.00E-06
rs9727115	1	99177253	99177253	2.00E-07
rs10190052	2	646674	646674	2.00E-07
rs11676855	2	235900171	235900171	9.00E-06
rs11677370	2	3841420	3841420	3.00E-06
rs13424957	2	165750390	165750390	3.00E-06
rs1515110	2	227122216	227122216	2.00E-07
rs1861612	2	230522398	230522398	7.00E-08
rs243021	2	60584819	60584819	3.00E-15
rs243088	2	60568745	60568745	3.00E-06
rs2681019	2	23187504	23187504	8.00E-08
rs2943640	2	227093585	227093585	7.00E-09
rs2943641	2	227093745	227093745	9.00E-12
rs3923113	2	165501849	165501849	1.00E-08
rs560887	2	169763148	169763148	9.00E-218
rs6546886	2	74245777	74245777	3.00E-06
rs6712932	2	105837598	105837598	6.00E-06
rs6723108	2	135479980	135479980	7.00E-08
rs693	2	21232195	21232195	9.00E-23

rs715	2	211543055	211543055	2.00E-147
rs7560163	2	151637936	151637936	7.00E-09
rs7578326	2	227020653	227020653	5.00E-20
rs7578597	2	43732823	43732823	1.00E-09
rs7593730	2	161171454	161171454	4.00E-08
rs780094	2	27741237	27741237	6.00E-53
rs815815	2	47399064	47399064	7.00E-07
rs925735	2	227179630	227179630	2.00E-08
rs10513788	3	182121573	182121573	5.00E-06
rs1107366	3	125904165	125904165	2.00E-06
rs1108842	3	52720080	52720080	1.00E-13
rs11128347	3	73619561	73619561	6.00E-07
rs11708067	3	123065778	123065778	7.00E-22
rs11717195	3	123082398	123082398	2.00E-08
rs11920090	3	170717521	170717521	8.00E-13
rs13081389	3	12289800	12289800	2.00E-07
rs1374910	3	185531661	185531661	1.00E-07
rs1470579	3	185529080	185529080	2.00E-19
rs1521418	3	116761660	116761660	8.00E-06
rs16861329	3	186666461	186666461	3.00E-08
rs16862964	3	187792711	187792711	7.00E-06
rs17036101	3	12277845	12277845	2.00E-07
rs1801282	3	12393125	12393125	6.00E-10
rs182052	3	186560782	186560782	5.00E-44
rs2063640	3	102203045	102203045	2.00E-06
rs2590838	3	52622086	52622086	2.00E-13
rs358806	3	55313400	55313400	3.00E-06
rs3773506	3	142431000	142431000	9.00E-06
rs4301033	3	150042618	150042618	6.00E-07
rs4402960	3	185511687	185511687	1.00E-17
rs4607103	3	64711904	64711904	1.00E-08
rs559356	3	34865597	34865597	3.00E-06
rs6769511	3	185530290	185530290	1.00E-09
rs6780569	3	23198484	23198484	4.00E-07
rs6808574	3	187740523	187740523	6.00E-09
rs6810075	3	186548565	186548565	2.00E-42
rs7612463	3	23336450	23336450	7.00E-09
rs7630877	3	179661318	179661318	7.00E-06
rs831571	3	64048297	64048297	8.00E-11
rs11096990	4	39286949	39286949	6.00E-07
rs12500138	4	166147712	166147712	6.00E-06
rs13434995	4	56467214	56467214	9.00E-06
rs1801214	4	6303022	6303022	3.00E-08
rs2055942	4	46968050	46968050	1.00E-06

rs3792615	4	164532801	164532801	9.00E-06
rs4458523	4	6289986	6289986	2.00E-09
rs4470583	4	162250932	162250932	4.00E-07
rs4689388	4	6270056	6270056	1.00E-08
rs6813195	4	153520475	153520475	4.00E-14
rs6815464	4	1309901	1309901	2.00E-20
rs6816344	4	73783404	73783404	1.00E-06
rs7656416	4	1254535	1254535	1.00E-08
rs7659604	4	122665514	122665514	9.00E-06
rs10461617	5	56104308	56104308	4.00E-06
rs12518099	5	89546109	89546109	7.00E-07
rs17053082	5	155394230	155394230	4.00E-07
rs17376456	5	93557702	93557702	3.00E-15
rs17823642	5	78341297	78341297	2.00E-09
rs2098713	5	37144574	37144574	3.00E-06
rs319598	5	134240235	134240235	2.00E-06
rs4457053	5	76424949	76424949	3.00E-12
rs6235	5	95728898	95728898	1.00E-26
rs6450176	5	53298025	53298025	7.00E-10
rs6595551	5	124139895	124139895	6.00E-06
rs6864869	5	89051857	89051857	3.00E-06
rs702634	5	53271420	53271420	7.00E-09
rs10440833	6	20688121	20688121	2.00E-22
rs10455872	6	161010118	161010118	5.00E-39
rs1048886	6	71289189	71289189	3.00E-08
rs10946398	6	20661034	20661034	1.00E-08
rs12214416	6	160910517	160910517	5.00E-08
rs1535500	6	39284050	39284050	2.00E-08
rs17057640	6	130019354	130019354	5.00E-06
rs3120139	6	160741622	160741622	4.00E-09
rs3132524	6	31136714	31136714	4.00E-09
rs3916765	6	32685550	32685550	1.00E-06
rs4273712	6	126964510	126964510	2.00E-13
rs4712523	6	20657564	20657564	7.00E-20
rs4712524	6	20657865	20657865	3.00E-10
rs4716055	6	9853919	9853919	4.00E-06
rs592423	6	139840693	139840693	4.00E-07
rs594442	6	93847884	93847884	1.00E-06
rs642858	6	140273647	140273647	2.00E-06
rs668459	6	139835689	139835689	9.00E-09
rs6930576	6	148704954	148704954	7.00E-07
rs6931514	6	20703952	20703952	2.00E-18
rs6937795	6	137291281	137291281	7.00E-06
rs7744392	6	35322763	35322763	3.00E-06

rs7754840	6	20661250	20661250	7.00E-16
rs7756992	6	20679709	20679709	2.00E-26
rs7766070	6	20686573	20686573	6.00E-11
rs7769051	6	133146796	133146796	2.00E-06
rs783147	6	161137990	161137990	3.00E-17
rs9295474	6	20652717	20652717	9.00E-06
rs9348440	6	20641336	20641336	3.00E-19
rs9362054	6	85178268	85178268	1.00E-06
rs9465871	6	20717255	20717255	3.00E-07
rs9470794	6	38106844	38106844	2.00E-10
rs9472138	6	43811762	43811762	7.00E-16
rs9502570	6	7258617	7258617	1.00E-09
rs998584	6	43757896	43757896	3.00E-15
rs1003247	7	35610162	35610162	6.00E-06
rs10229583	7	127246903	127246903	2.00E-10
rs10954361	7	131815343	131815343	7.00E-06
rs1525739	7	16889812	16889812	6.00E-06
rs17168486	7	14898282	14898282	3.00E-07
rs17364464	7	22514053	22514053	4.00E-06
rs1799884	7	44229068	44229068	2.00E-22
rs2191349	7	15064309	15064309	3.00E-44
rs2284219	7	30714436	30714436	8.00E-06
rs4607517	7	44235668	44235668	7.00E-92
rs6467136	7	127164958	127164958	5.00E-11
rs741301	7	36917995	36917995	8.00E-06
rs7636	7	100490077	100490077	5.00E-06
rs7791362	7	8142927	8142927	4.00E-06
rs7795991	7	13900731	13900731	7.00E-07
rs7800418	7	26606005	26606005	7.00E-07
rs791595	7	127862802	127862802	3.00E-13
rs849134	7	28196222	28196222	3.00E-09
rs849135	7	28196413	28196413	2.00E-09
rs864745	7	28180556	28180556	5.00E-14
rs972283	7	130466854	130466854	2.00E-10
rs11558471	8	118185733	118185733	3.00E-20
rs13266634	8	118184783	118184783	2.00E-14
rs1561927	8	129568078	129568078	1.00E-07
rs17232789	8	122622500	122622500	6.00E-06
rs17428041	8	21711431	21711431	2.00E-07
rs2077233	8	138194869	138194869	7.00E-06
rs2116081	8	125697673	125697673	6.00E-06
rs2439312	8	32412359	32412359	7.00E-06
rs2648875	8	129072161	129072161	2.00E-06
rs2980879	8	126481475	126481475	1.00E-09

rs328	8	19819724	19819724	2.00E-28
rs3802177	8	118185025	118185025	2.00E-18
rs4527850	8	134196849	134196849	2.00E-06
rs515071	8	41519462	41519462	1.00E-08
rs516946	8	41519248	41519248	2.00E-07
rs7845219	8	95937502	95937502	6.00E-08
rs896854	8	95960511	95960511	1.00E-09
rs10811661	9	22134094	22134094	1.00E-27
rs10814916	9	4293150	4293150	6.00E-12
rs10965250	9	22133284	22133284	1.00E-10
rs10980508	9	113419759	113419759	1.00E-06
rs10993738	9	93633240	93633240	5.00E-06
rs11787792	9	139252148	139252148	2.00E-10
rs1327796	9	112526289	112526289	3.00E-06
rs13292136	9	81952128	81952128	3.00E-08
rs1333051	9	22136489	22136489	6.00E-10
rs1421001	9	90159313	90159313	4.00E-06
rs17584499	9	8879118	8879118	9.00E-10
rs17791513	9	81905590	81905590	3.00E-08
rs2383208	9	22132076	22132076	2.00E-29
rs2796441	9	84308948	84308948	2.00E-06
rs4978848	9	112521126	112521126	3.00E-06
rs564398	9	22029547	22029547	1.00E-06
rs649891	9	10430602	10430602	6.00E-06
rs651007	9	136153875	136153875	2.00E-82
rs6560517	9	79038170	79038170	7.00E-06
rs7018475	9	22137685	22137685	3.00E-08
rs7020996	9	22129579	22129579	2.00E-07
rs7034200	9	4289050	4289050	1.00E-13
rs7041847	9	4287466	4287466	2.00E-14
rs773506	9	93975471	93975471	6.00E-06
rs824248	9	28772700	28772700	8.00E-06
rs10510110	10	124192430	124192430	1.00E-07
rs10741243	10	132947962	132947962	5.00E-06
rs10788575	10	89768584	89768584	9.00E-06
rs10885122	10	113042093	113042093	3.00E-16
rs10885531	10	115814392	115814392	4.00E-06
rs10886471	10	121149403	121149403	7.00E-09
rs10906115	10	12314997	12314997	1.00E-08
rs1111875	10	94462882	94462882	3.00E-19
rs11257655	10	12307894	12307894	3.00E-09
rs12219125	10	20593087	20593087	9.00E-09
rs12571751	10	80942631	80942631	2.00E-10
rs12779790	10	12328010	12328010	1.00E-10

rs1571942	10	20542634	20542634	3.00E-07
rs1802295	10	70931474	70931474	4.00E-08
rs2812533	10	71452285	71452285	5.00E-06
rs4462262	10	59189178	59189178	9.00E-08
rs4506565	10	114756041	114756041	5.00E-12
rs4838605	10	49699957	49699957	2.00E-09
rs5015480	10	94465559	94465559	1.00E-15
rs531676	10	99637578	99637578	9.00E-06
rs6583826	10	94347830	94347830	7.00E-06
rs7901695	10	114754088	114754088	1.00E-48
rs7903146	10	114758349	114758349	8.00E-75
rs10501320	11	47293799	47293799	1.00E-88
rs10830962	11	92698427	92698427	1.00E-16
rs10830963	11	92708710	92708710	6.00E-175
rs10838687	11	47312892	47312892	7.00E-12
rs11023332	11	14784110	14784110	7.00E-06
rs11212617	11	108283161	108283161	3.00E-09
rs11603334	11	72432985	72432985	3.00E-102
rs11605924	11	45873091	45873091	4.00E-15
rs1387153	11	92673828	92673828	2.00E-36
rs1552224	11	72433098	72433098	1.00E-22
rs163182	11	2844216	2844216	2.00E-17
rs163184	11	2847069	2847069	2.00E-14
rs174541	11	61565908	61565908	3.00E-09
rs174550	11	61571478	61571478	4.00E-274
rs2166706	11	92691532	92691532	2.00E-09
rs2237892	11	2839751	2839751	2.00E-42
rs2237895	11	2857194	2857194	1.00E-09
rs2237897	11	2858546	2858546	1.00E-16
rs231362	11	2691471	2691471	3.00E-13
rs2722769	11	11228374	11228374	2.00E-06
rs3842770	11	2178670	2178670	3.00E-08
rs5215	11	17408630	17408630	3.00E-11
rs5219	11	17409572	17409572	7.00E-11
rs7107217	11	129473690	129473690	3.00E-07
rs7111546	11	22829757	22829757	2.00E-06
rs712022	11	22843155	22843155	6.00E-06
rs7944584	11	47336320	47336320	2.00E-18
rs7945071	11	110243922	110243922	7.00E-06
rs8181588	11	2831541	2831541	5.00E-09
rs9300039	11	41915366	41915366	6.00E-08
rs10842994	12	27965150	27965150	8.00E-06
rs11066453	12	113365621	113365621	6.00E-44
rs1153188	12	55098996	55098996	2.00E-07

rs11615866	12	5393329	5393329	1.00E-06
rs12229654	12	111414461	111414461	9.00E-58
rs12304921	12	51357542	51357542	7.00E-06
rs12427353	12	121426901	121426901	4.00E-06
rs1495377	12	71577101	71577101	2.00E-06
rs1531343	12	66174894	66174894	4.00E-09
rs1727313	12	123640853	123640853	1.00E-08
rs2074356	12	112645401	112645401	3.00E-126
rs2261181	12	66212318	66212318	4.00E-08
rs2358944	12	66117558	66117558	4.00E-06
rs2657888	12	56938383	56938383	8.00E-06
rs35767	12	102875569	102875569	2.00E-09
rs3741489	12	133417802	133417802	2.00E-06
rs4760790	12	71634794	71634794	4.00E-06
rs499368	12	320920	320920	8.00E-13
rs601339	12	123174743	123174743	4.00E-06
rs6488898	12	124203832	124203832	3.00E-10
rs7305618	12	121402932	121402932	1.00E-08
rs7955516	12	20498036	20498036	4.00E-08
rs7957197	12	121460686	121460686	2.00E-08
rs7961581	12	71663102	71663102	1.00E-09
rs10507349	13	26781528	26781528	2.00E-07
rs1359790	13	80717156	80717156	6.00E-09
rs16953622	13	97508739	97508739	7.00E-06
rs2038823	13	96951433	96951433	5.00E-11
rs9552911	13	23864657	23864657	2.00E-08
rs1009170	14	92636713	92636713	2.00E-06
rs17244419	14	97171075	97171075	8.00E-06
rs4904947	14	92972162	92972162	8.00E-06
rs730570	14	101142890	101142890	8.00E-06
rs11071657	15	62433962	62433962	4.00E-08
rs11630316	15	70369378	70369378	9.00E-06
rs11634397	15	80432222	80432222	2.00E-09
rs12899811	15	91544076	91544076	6.00E-07
rs1436953	15	62414014	62414014	8.00E-06
rs1436955	15	62404382	62404382	7.00E-07
rs1549318	15	71109147	71109147	2.00E-10
rs2028299	15	90374257	90374257	2.00E-11
rs4502156	15	62383155	62383155	4.00E-20
rs4777845	15	93877425	93877425	9.00E-06
rs7119	15	77777632	77777632	5.00E-07
rs7163757	15	62391608	62391608	4.00E-06
rs7172432	15	62396389	62396389	9.00E-14
rs7178572	15	77747190	77747190	2.00E-11

rs7403531	15	38822905	38822905	4.00E-09
rs8025118	15	79552379	79552379	3.00E-06
rs8042680	15	91521337	91521337	2.00E-10
rs11642841	16	53845487	53845487	3.00E-08
rs12051272	16	82663288	82663288	6.00E-48
rs16955379	16	81489373	81489373	3.00E-07
rs17177078	16	24810681	24810681	5.00E-06
rs17797882	16	79406918	79406918	9.00E-07
rs1800775	16	56995236	56995236	3.00E-93
rs2925979	16	81534790	81534790	3.00E-21
rs74019828	16	58209274	58209274	5.00E-08
rs8050136	16	53816275	53816275	1.00E-47
rs8052123	16	57563671	57563671	5.00E-06
rs9921518	16	54494424	54494424	9.00E-06
rs9936385	16	53819169	53819169	1.00E-12
rs9939609	16	53820527	53820527	4.00E-51
rs1859962	17	69108753	69108753	2.00E-16
rs231513	17	41965200	41965200	8.00E-06
rs312457	17	6940393	6940393	8.00E-13
rs391300	17	2216258	2216258	3.00E-09
rs4430796	17	36098040	36098040	1.00E-11
rs4790333	17	2262703	2262703	3.00E-09
rs507506	17	7118322	7118322	2.00E-06
rs623323	17	700020	700020	4.00E-06
rs75493593	17	6945087	6945087	5.00E-15
rs10460009	18	2948029	2948029	9.00E-06
rs12970134	18	57884750	57884750	5.00E-13
rs7243299	18	7755771	7755771	8.00E-06
rs8090011	18	7068462	7068462	8.00E-09
rs8098064	18	8209269	8209269	4.00E-06
rs1423096	19	7739177	7739177	1.00E-07
rs3786897	19	33893008	33893008	1.00E-08
rs4420638	19	45422946	45422946	2.00E-178
rs472265	19	39580737	39580737	9.00E-06
rs4805885	19	33906123	33906123	2.00E-08
rs731839	19	33899065	33899065	8.00E-12
rs8108269	19	46158513	46158513	5.00E-06
rs328506	20	56029604	56029604	2.00E-06
rs4812829	20	42989267	42989267	3.00E-10
rs4814615	20	17357573	17357573	5.00E-06
rs6017317	20	42946966	42946966	1.00E-11
rs6047116	20	20848968	20848968	2.00E-06
rs926392	20	37690464	37690464	5.00E-07
rs1452093	21	28744356	28744356	2.00E-06

rs2833610	21	33385186	33385186	4.00E-06
rs9977499	21	28734997	28734997	4.00E-06
rs16983214	22	27343454	27343454	2.00E-06
rs2106294	22	31645759	31645759	4.00E-06
rs2412980	22	30592069	30592069	4.00E-06
rs470089	22	44248504	44248504	9.00E-06
rs12010175	23	152862638	152862638	2.00E-09
rs5945326	23	152899922	152899922	7.00E-16

Table S2.The iTEA, Haploreg and RegulomeDB scores of the 11 literature-validated SNPs.

Literature-validated SNPs	iTEA (1: functional)	RegulomeDB Score*	Haploreg Number of Motifs Changed
rs11257655	0	4	5 altered motifs
rs11603334	0	2b	1 altered motifs
rs1535500	0	5	5 altered motifs
rs340874	0	4	1 altered motifs
rs4506565	0	7	2 altered motifs
rs780094	0	2c	9 altered motifs
rs7903146	0	5	7 altered motifs
rs10946398	1	5	5 altered motifs
rs2074356	1	3a	1 altered motifs
rs35767	1	3a	7 altered motifs
rs4607517	1	5	7 altered motifs

*In Regulome DB score, 2b and 2c means “likely to affect binding”, and 3a means “less likely to affect binding”.

Table S3. The RegulomeDB and Haploreg scores of the nine iTEA-identified SNPs.

iTEA	RegulomeDB Score*	Haploreg.number of motifs changed
rs10946398	5	5 altered motifs
rs1107366	7	3 altered motifs
rs2074356	3a	1 altered motifs
rs2796441	2b	23 altered motifs
rs35767	3a	7 altered motifs
rs4607517	5	7 altered motifs
rs6930576	7	3 altered motifs
rs6937795	4	6 altered motifs
rs815815	4	6 altered motifs

*In Regulome DB score, 2b and 2c means “likely to affect binding”, and 3a means “less likely to affect binding”.

Table S4. The identified response elements of the nine functional SNP candidates from iTEA.

SNPID	width	seqMatch
rs10946398	8	ttAtgctg
rs10946398	10	gttAtgctgt
rs1107366	12	cactgtaAagga
rs1107366	10	tgtaAaggaa
rs1107366	8	gtaAagga
rs1107366	10	cactgtaAag
rs1107366	20	ccccagaaactcactgtaAa
rs2074356	10	cttcagatGt
rs2074356	10	gatGtgaact
rs2074356	12	acttcagatGtg
rs2074356	8	agatGtga
rs2074356	10	ttcagatGtg
rs2074356	8	ttcagatG
rs2074356	10	cttcagatGt
rs2074356	10	ttcagatGtg
rs2074356	10	tcagatGtga
rs2074356	10	agatGtgaac
rs2074356	10	cttcagatGt
rs2074356	10	agatGtgaac
rs2796441	12	ttgctgtgaGtt
rs2796441	12	gctgtgaGttgc
rs2796441	10	gctgtgaGtt
rs2796441	12	gaGttgctaatt
rs2796441	10	gtgaGttgct
rs35767	10	ttttccAcat
rs35767	8	tccAcatg
rs35767	12	tttccAcatgac
rs35767	10	ttccAcatga
rs35767	10	tttttccAca
rs35767	10	tccAcatgac
rs35767	12	ttttccAcatga
rs35767	10	tttttccAca
rs35767	10	tccAcatgac
rs35767	10	ccAcatgact
rs35767	10	ttccAcatga
rs35767	10	tccAcatgac
rs35767	8	tccAcatg
rs35767	10	tttttccAca
rs35767	10	ttttccAcat
rs35767	12	tccAcatgactc
rs35767	10	tttttccAca

rs4607517	10	gttgGgtgac
rs4607517	10	gttgGgtgac
rs4607517	12	ttgGgtgacagc
rs4607517	12	ttgGgtgacagc
rs4607517	10	ttgGgtgaca
rs4607517	10	ttgGgtgaca
rs4607517	12	tgGgtgacagct
rs6930576	10	gaaaccGatt
rs6930576	10	aaccGattgt
rs6930576	12	accGattgttga
rs6937795	8	taagcAca
rs6937795	10	ttaagcAcat
rs6937795	8	attaagcA
rs815815	10	aagCagttta
rs815815	8	agCagttt
rs815815	10	aagCagttta
rs815815	10	gaaagCagtt
